# The Roma Population Living in Segregated Settlements in Eastern Slovakia Has a Higher Prevalence of Metabolic Syndrome, Kidney Disease, Viral Hepatitis B and E, and Some Parasitic Diseases Compared to the Majority Population

**DOI:** 10.3390/ijerph17093112

**Published:** 2020-04-29

**Authors:** Zelmira Macejova, Pavol Kristian, Martin Janicko, Monika Halanova, Sylvia Drazilova, Daniela Antolova, Maria Marekova, Daniel Pella, Andrea Madarasova-Geckova, Peter Jarcuska, HepaMeta Team

**Affiliations:** 1 1st Department of Internal Medicine, Faculty of Medicine, Pavol Jozef Safarik University and Louis Pasteur University Hospital, 040 11 Kosice, Slovakia; zelmira.macejova@upjs.sk; 2 Department of Infectology and Travel Medicine, Faculty of Medicine, Pavol Jozef Safarik University and Louis Pasteur University Hospital, 040 01 Kosice, Slovakia; 3 2nd Department of Internal Medicine, Faculty of Medicine, Pavol Jozef Safarik University and Louis Pasteur University Hospital, 040 11 Kosice, Slovakia; martin.janicko1@upjs.sk (M.J.); peter.jarcuska@upjs.sk (P.J.); 4 Department of Epidemiology, Faculty of Medicine, Pavol Jozef Safarik University, 040 01 Kosice, Slovakia; monika.halanova@upjs.sk; 5 Department of Internal Medicine, Hospital Poprad and Faculty of Medicine, Pavol Jozef Safarik University, 058 01 Poprad, Slovakia; drazilova.s@nemocnicapp.sk; 6 Department of Parasitic Diseases, Institute of Parasitology SAS, Hlinkova 3, 040 01 Kosice, Slovakia; antolova@saske.sk; 7 Department of Medical and Clinical Biochemistry, Faculty of Medicine, Pavol Jozef Safarik University, 040 11 Kosice, Slovakia; maria.marekova@upjs.sk; 8 2nd Department of Cardiology, Faculty of Medicine, Pavol Jozef Safarik University and East Slovak, Institute of Cardiovascular Diseases, Faculty of Medicine, Pavol Jozef Safarik University, 040 11 Kosice, Slovakia; daniel.pella@upjs.sk; 9 Department of Health Psychology, Faculty of Medicine, Pavol Jozef Safarik University, 040 11 Kosice, Slovakia; andrea.geckova@upjs.sk

**Keywords:** Roma population, metabolic syndrome, hepatitis B, hepatitis E, *Trichinella* spp., *Toxocara* spp.

## Abstract

*Background*: The Roma population is one of the largest marginalized population groups in Europe. The aim of our work was to summarize the morbidity of lifestyle-related diseases and infectious diseases in the Roma population living in segregated settlements. *Methods*: We used data from the cross-sectional study HepaMeta, in which we examined 452 Roma subjects with an average age of 34.7 ± 9.1 years, 35.2% of which were men, and 403 non-Roma subjects with an average age of 33.5 ± 7.4 years, 45.9% of which were men. We collected data by means of a questionnaire, anthropometric measures, and we analyzed blood and urine samples. *Results*: Roma subjects had a higher incidence of metabolic syndrome (RR: 1.478 (1.159–1.885), *p* < 0.0001), obesity or waist circumference >94 cm in men/80 cm in women (RR: 1.287 (1.127–1.470), *p* < 0.0001), and HDL-C < 1.03 mmol/L in men or <1.29 in women (RR: 2.004 (1.730–2.321), *p* < 0.0001) than their non-Roma counterparts. Subjects of the Roma population were more frequently diagnosed with kidney disease (RR: 1.216 (1.096–1.349), *p* < 0.0001), HBsAg positivity (RR: 4.468 (2.373–8.415), *p* < 0.0001), anti HBc IgG positivity (RR: 3.13 (2.598–4.224), *p* < 0.0001), and anti HEV positivity (RR: 2.972 (1.226–7.287), *p* < 0.0001). Serological markers of *Toxoplasma gondii* infection and *Toxocara* spp. were observed much more frequently among Roma than non-Roma subjects (RR: 1.868 (1.520–2.296), *p* < 0.0001, for *Toxoplasma gondii*; and RR: 21.812 (8.097–58.761), *p* < 0.0001, for *Toxocara* spp.). *Conclusions*: Poor socio-economic conditions, an unhealthy lifestyle, and barriers precluding access to healthcare are factors that affect the Roma population in settlements and lead to an increased prevalence of metabolic syndrome and its components, kidney disease, viral hepatitis B and E, and some parasitic diseases.

## 1. Introduction

The Roma population is one of the oldest and largest minorities. The Roma, mainly due to their nomadic way of life in the past centuries, do not have their own state and live in scattered groups in several European countries. There are approximately 400,000 Roma people living in the Slovak Republic, which accounts for about 7.5% of the Slovak population. However, in the official census, only a small number of them claim their Roma nationality.

Approximately one sixth of the Roma population in Slovakia live in segregated Roma settlements [1]. The largest part of the Roma population in Slovakia lives in the region of Eastern Slovakia [2]. Roma living in settlements have disastrous socio-economic conditions. More than 80% of them possess only basal education, over 90% of them are unemployed, and their financial income is significantly lower than the majority of the Slovak population. One sixth of the population in Roma settlements have no access to electricity and only about half of this population has a stable water supply, bathrooms, showers, or toilets [3]. Approximately half of the Roma population in settlements have poor access to healthcare, which mainly stems from the poor socio-economic situation of this population, but also out of their distrust of official providers of healthcare, and their reliance on self-medication [4]. Roma are less likely to visit a general practitioner and are less likely to attend check-ups compared to the majority population [5]. Roma suffer more often from communicable and non-communicable disease and have a shorter life expectancy than national averages [6,7].

The aim of our work was to compare the incidence of infectious and lifestyle-related diseases between the Roma population living in the settlements and the majority population of Eastern Slovakia.

## 2. Materials and Methods

We used data from the cross-sectional study HepaMeta conducted in 2011 in the Eastern Slovakia region. We studied subjects in the age range of 18–55 years in the Roma population living in settlements, while the control group was from the majority population and were of the same age range. The majority population was divided into 2 subgroups: 46% of the subjects lived, and 54% of which did not live, near Roma settlements. The prevalence of communicable and non-communicable diseases was compared between the Roma and the majority non-Roma population.

Subjects filled in a questionnaire containing data on education, socio-economic status, and toxicological history, including intravenous drug use, toluene sniffing, smoking, and drinking alcohol.

All subjects underwent an anthropometric examination: weight, height, waist, and hip circumferences, as well as blood pressure measurement in resting conditions. The BMI (body mass index) was calculated from height and weight measurements. Obesity was defined as a BMI ≥ 30 kg/m^2^, and underweight as BMI < 18.5 kg/m^2^ [8,9]. Metabolic syndrome (MetS) had been defined as a waist circumference >94 cm for males and >80 cm for females or a BMI > 30 kg/m^2^ and any two of the following factors:

- A raised level of triglycerides (TG) ≥ 150 mg/dL (1.7 mmol/L), or specific treatment for this lipid abnormality.
- A reduced level of high-density lipoprotein cholesterol (HDL-C) < 40 mg/dL (1.03 mmol/L) in males, <50 mg/dL (1.29 mmol/L) in females, or specific treatment for this lipid abnormality.
- Elevated blood pressure, systolic ≥ 130 or diastolic ≥ 85 mmHg, or treatment of previously diagnosed hypertension.
- A raised fasting plasma glucose ≥ 100 mg/dL (5.6 mmol/L), or previously diagnosed type 2 diabetes mellitus (T2DM) [10].

Blood and urine samples were collected from the subjects during the examination. In the plasma we examined glycemia, creatinine, total cholesterol, HDL-C, LDL-C (low-density lipoprotein cholesterol), TG, and uric acid. In urine we examined creatinine levels and proteinuria. The glomerular filtration rate (GFR) was calculated using the MDRD (modification of diet in renal disease) equation based on serum creatinine from a morning, fasting serum sample [11].

A detailed description of the study design and the biochemical methods used is presented in the work of Madarasova-Geckova et al. (2014) [2].

HBsAg (hepatitis B surface antigen), anti-HBc (antibodies to the hepatitis core antigen) IgG (immunoglobulin G), and anti-HCV (antibodies to hepatitis C) testing was performed by Enzygnost (Siemens, Eschborn, Germany) [2,12]. Detection of anti-HEV (antibodies to hepatitis E) was done using a commercial enzyme-linked immunosorbent assay (ELISA) kit (DRG Instruments GmbH, Marburg, Germany) [13]. The prevalence of *Chlamydia trachomatis* was examined by directly elucidating the presence of the pathogen via a polymerase chain reaction (PCR) using the commercial DNA-sorb-AM nucleic acid extraction kit and the AmpliSens^®^ *Chlamydia trachomatis*-EPh PCR kit (the Federal Budget Institution of Science, Moscow, Russia) [14]. Detection of antibodies to *Toxocara* spp., *Trichinella* spp., *Echinococcus multilocularis*, *Echinococcus granulosus*, and *Toxoplasma gondii* are described in detail in previously published manuscripts [15,16,17].

An elevated blood pressure was defined as a systolic blood pressure ≥ 130 or a diastolic blood pressure ≥ 85 mmHg, or treatment of a previously diagnosed hypertension. An impaired fasting glucose (IFG) was defined as a fasting glucose level between 5.6 and 6.99 mmol/L, while T2DM was defined as fasting glucose level ≥ 7.0 mmol/L. Men with hyperuricemia had a uric acid level > 420 μmol/L while women had levels > 390 μmol/L.

Kidney disease was defined as a history of renal disease, or the presence of proteinuria/hematuria, or a GFR < 60 mL/min [18].

The study was approved by the Ethics Committee of P.J. Safarik University, Faculty of Medicine in Kosice, Slovakia. Participation in the study was fully voluntary and anonymous. Detailed information about the study and its procedures was given to all respondents, and an informed consent was obtained prior to the medical examination.

### Statistical Analysis

Values are presented as absolute and relative counts. Differences in values were also quantified as relative risks from 2 × 2 contingency tables with 95% confidence intervals. Statistical significance was tested by either a Fisher exact test in case of 2 × 2 tables, or chi-square in case of more than two categories.

## 3. Results

The study enrolled 452 Roma subjects with an average age of 34.7 ± 9.1 years, 35.2% of which were men, and 403 non-Roma with an average age of 33.5 ± 7.4 years, 45.9% of which were men. The characteristics of the patient population are shown in Table 1.

**Figure 1:**
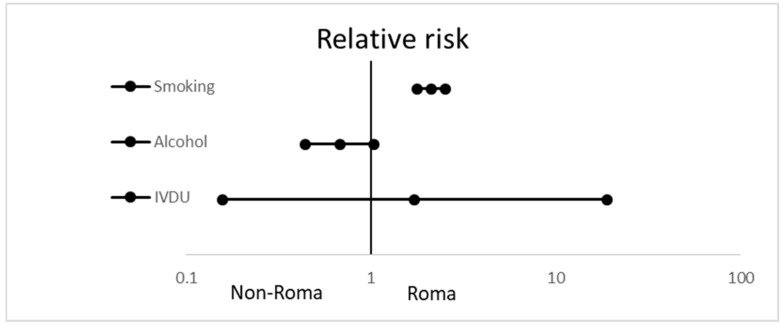
Relative risk with 95% confidence intervals of smoking, significant alcohol consumption, and intravenous drug use in Roma participants, compared with the non-Roma population.

Figure 1 shows the consumption of toxic products in the Roma and the non-Roma majority population. Intravenous drug use was sporadic in both groups; the differences were not significant. A total of 7.6% of Roma and 11.2% of the non-Roma reported hazardous alcohol consumption (>20 g per day) in the questionnaire. The difference between the two groups was not significant. A total of 59.8% of Roma and 28.2% of non-Roma were smokers (RR: 2.119 (1.778–2.526); *p* < 0.0001).

Toluene sniffing was prevalent in 2.2% of the Roma and in none of the non-Roma population; the difference was not significant.

**Figure 2:**
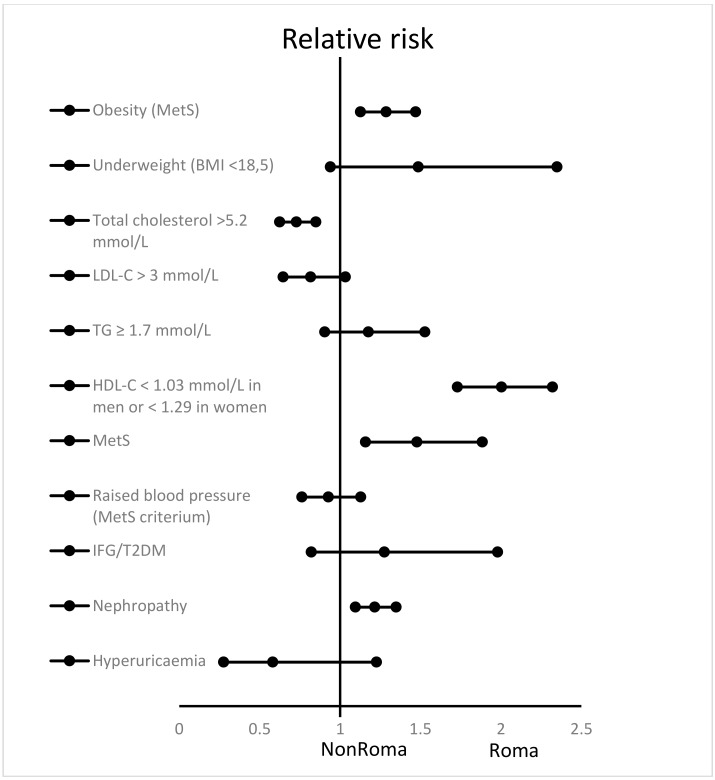
Relative risk with 95% confidence intervals of common non-communicable diseases and certain risk factors in Roma participants, compared with the non-Roma population. Obesity was defined as in the international diabetes foundation (IDF) 2006 MetS definition. LDL-C—low-density lipoprotein cholesterol; TG—triglycerides; HDL-C—high-density lipoprotein cholesterol; MetS—metabolic syndrome; IFG—impaired fasting glucose; T2DM—type 2 diabetes mellitus.

Figure 2 describes the incidence of lifestyle-related diseases in the Roma and non-Roma majority population. A total of 58.9% of Roma and 45.8% of non-Roma showed obesity or a waist circumference > 94 cm in men/80 cm in women (RR: 1.287 (1.127–1.470); *p* < 0.0001). Underweight was found in 10% of Roma and 6.7% of non-Roma; the difference was not significant. Total cholesterol > 5.2 mmol/L was found in 37.4% of Roma and 51.4% of non-Roma; the difference was significant (RR: 0.729 (0.625–0.850); *p* < 0.0001). LDL-C value > 3.0 mmol/L and triglycerides > 1.7 mmol/L occurred almost equally in both the Roma and non-Roma populations. Low HDL-C, which is considered to be one of the criteria for the diagnosis of metabolic syndrome (<1.03 mmol/L in men or <1.29 in women) was found in 70% of Roma, but only in 34.9% of non-Roma; the difference was significant (RR: 2004 (1730–2321); *p* < 0.0001). Metabolic syndrome was diagnosed in 29.6% of Roma and 20.1% of non-Roma (RR: 1.478 (1.159–1.885); *p* < 0.0001). There was no significant difference between Roma and non-Roma in the occurrence of elevated blood pressure, IFG, T2DM, or hyperuricemia. Nephropathy was more frequently observed in the Roma population compared to the non-Roma population (RR: 1.216 (1.096–1.349); *p* < 0.0001).

**Figure 3:**
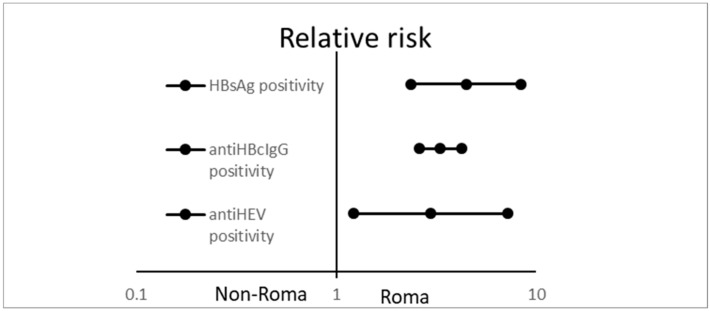
Relative risk with 95% confidence intervals of hepatitis B and hepatitis E serological markers positivity in Roma participants, compared with the non-Roma population.

Figure 3 shows the incidence of viral hepatitis in Roma and non-Roma. Hepatitis B markers were diagnosed with a significantly higher frequency in Roma living in segregated settlements: HBsAg positivity was found in 12.4% of Roma and 2.8% of non-Roma (RR: 4.468 (2.373–8.415); *p* < 0.0001) and anti-HBc IgG positivity in 52.8% of Roma and 15.9% of the non-Roma population (RR: 3.13 (2.598–4.224); *p* < 0.0001). Antibodies against hepatitis E were found in a significantly higher frequency in Roma (21.5%) than in the non-Roma population (7.2%) (RR: 2.972 (1.226–7.287); *p* < 0.0001.

Chronic hepatitis C was found only in 0.7% of subjects in the Roma population and HCV was not diagnosed in the majority non-Roma population; the difference was not significant.

**Figure 4:**
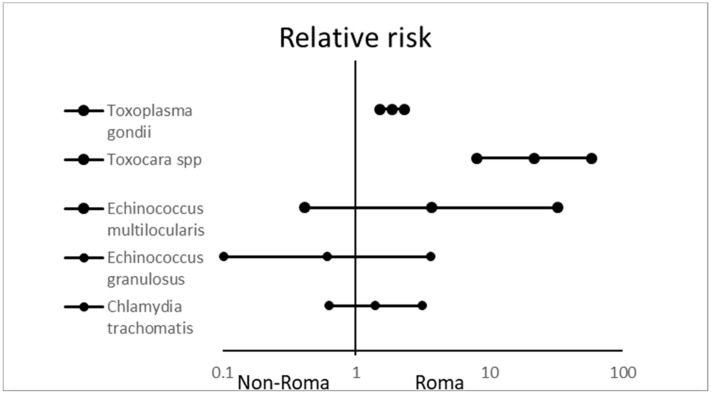
Relative risk with 95% confidence intervals of serological markers positivity of selected parasites and *Chlamydia trachomatis* in Roma participants, compared with the non-Roma population.

Figure 4 describes the incidence of some parasitic diseases and *Chlamydia trachomatis* infections in the Roma and non-Roma communities. Serological markers of *Toxoplasma gondii* infection were significantly more frequent among Roma than non-Roma people (45% vs. 24.1%; RR: 1.868 (1.520–2.296); *p* < 0.0001). Antibodies to *Toxocara* spp. were detected in 22.1% of Roma and only 1% of non-Roma; the difference was significant (RR: 21.812 (8.097–58.761); *p* < 0.0001).

Antibodies against *Trichinella* spp., *Echinococcus granulosus*, *Echinococcus multilocularis*, and *Chlamydia trachomatis* were sporadic in both groups (antibodies to *Trichinella* spp. in the non-Roma population were not detected at all); the differences were not significant.

## 4. Discussion

In the HepaMeta study we observed a higher prevalence of MetS, some viral hepatitis, and some parasitic diseases in the Roma population aged between 18 and 55 living in segregated settlements compared to the majority non-Roma population in Eastern Slovakia. Out of the individual parameters of metabolic syndrome, obesity and/or a waist circumference >94 cm in men and >80 cm in women were more common in the Roma population. Low levels of HDL-C also occurred more frequently in the Roma population as another parameter of MetS. Other parameters of metabolic syndrome (IFG or T2DM, hypertriglyceridemia, and elevated blood pressure) were not observed to be more prevalent in the Roma population than in the majority non-Roma population. These findings may correlate with the lifestyle of the Roma population, but could also be affected by genetic predisposition.

Roma men and women have less physical activity at work compared to the majority non-Roma population; Roma women less often practice brisk walks or sports. On the other hand, Roma men and women dance more often than non-Roma people [19]. Consumption of fruit, vegetables, and dairy products (related to poverty in Slovakia) is significantly lower in Roma people living in settlements compared to the majority population. Roma women eat meat and drink soft drinks more often than women from the majority population; these eating habits can increase MetS prevalence [20]. The Roma population in the Czech Republic has similar eating habits. An analysis of Roma nutritional habits revealed that their energy intake is less than 50% carbohydrates and more than 30% fats, which may be considered as excessive caloric intake [21,22]. Most of the Roma population with obesity or overweight usually have pertinent weight problems since childhood, and when it comes to weight reduction, they prefer a plan drawn up by a doctor, and not self-management [23].

Obesity in the Roma population is likely to be due to an unhealthy lifestyle. Differences in the prevalence of obesity between Roma and non-Roma could not be explained by their distinct genetic susceptibility [24,25].

Low HDL-C levels occur more than twice as often in the Roma population as opposed to the majority non-Roma population in our study group. In addition to lifestyle, HDL-C levels in the Roma are fundamentally influenced by the different genetics of the Roma population compared to the majority non-Roma, as confirmed by several genetic studies from Hungary [26,27,28,29].

The prevalence of IFG or T2DM in the Roma population in Eastern Slovakia was comparable to that of the majority non-Roma population; this finding was probably influenced by the relatively young age of both groups of participants. Hungarian authors have observed a population a decade older, where the prevalence of IFG or T2DM was significantly higher in the Roma population compared to the majority non-Roma. The finding was related to the Roma lifestyle, considering the fact that the majority non-Roma population carried a greater number of risk alleles for T2DM compared to their Roma counterparts [30].

Summarizing our results and the literature data, we can conclude that MetS is more common in the Roma than in the general population. Obesity, which occurs at a younger age, is involved in its development, while it is associated with IFG or T2DM at a later age. Obesity and diabetes develop in the Roma population due to an unhealthy lifestyle (lack of exercise, poor diet). Genetic factors for obesity and diabetes are not substantially different in comparison with the majority. Another criterion of MetS, which occurs much more frequently in the Roma population than in the majority population, is low HDL-C. The genetic predisposition in the Roma population is also fundamentally involved in this finding.

In the HepaMeta study, we did not observe any significant differences in the incidence of hyperuricemia between Roma and non-Roma populations. Although Roma have a more frequent MetS, the average uric acid values in the Roma population were significantly lower than that of the majority non-Roma population [31].

Roma patients with MetS have higher levels of hs-CRP (high sensitivity C-reactive protein) and GGT (gamma-glutamyl transpeptidase), which is a surrogate marker for fatty liver disease [31,32,33].

MetS is associated with an increased cardiovascular risk [34]. Another factor that increases cardiovascular risk is smoking. The number of smokers in the Roma population is significantly higher than in the majority population. Roma subjects usually start smoking at a younger age when compared to the majority population [35]. The increased prevalence of smoking in the Roma does not seem to have a genetic background [36]. Cardiovascular risk is very high in the Roma population [37,38]. However, in the published literature, data on the prevalence of cardiovascular morbidity and mortality in the Roma population are absent.

Roma subjects have a higher incidence of kidney disease in the HepaMeta study compared to the majority non-Roma population; the cause is not clearly identified. Chronic hepatitis B infection, which is several times more common in the Roma population than in the non-Roma population, leads to the occurrence of chronic glomerulonephritis in some patients. Some diseases that lead to chronic kidney damage, such as Alport syndrome, are more common in the Roma population as well [39]. Roma in Slovakia have a 34% higher risk of developing end-stage renal disease [40]. The average age at enrollment in the hemodialysis program is significantly lower in the Roma population than in the majority population. In Croatia, the average age at the start of dialysis for Roma is 29 years, in the majority non-Roma it is at 67 years. Roma have the same chances for inclusion in the dialysis program as other patients with kidney disease, but the problem is noncompliance in pre-transplant examinations as well as post-kidney transplantation [41]. Roma kidney transplant recipients are at a higher risk of mortality and worse graft outcomes compared to the majority population [42].

Some studies have been published examining the ethnic genetic diversity of the Roma population in Europe. Many of the Roma diasporas have remained in relative isolation over the centuries, leading to a high degree of inbreeding and consanguinity [43]. The result of this is a high incidence of hereditary disorders [44]. Some congenital diseases (e.g., congenital glaucoma, congenital hypothyroidism, short-chain acyl-CoA dehydrogenase deficiency) are more common in the Slovak Roma population than in the majority non-Roma population [45,46,47].

In the HepaMeta study, Roma and non-Roma patients aged between 18 and 55 were enrolled. Patients in both groups were not vaccinated against hepatitis B. HBsAg positivity (maker of chronic hepatitis B virus infection) was more frequent in the Roma population living in settlements compared to that of the majority population. A similar situation is observed for anti-HBc IgG positivity that is present in either chronic hepatitis B virus infection or after recovery from an acute hepatitis B infection. A overall higher prevalence of hepatitis B virus infection was observed in the Roma population in both children and adults when compared to the majority population and has been described in several studies [48,49,50].

The prevalence of chronic hepatitis B infection in Roma settlements in the HepaMeta study is alarming. An approximately equal high prevalence is observed in communities of migrants from southeast Asia living in the United States of America [51]. In the HepaMeta study, the Roma population had more risk factors for the transmission of infectious hepatitis B, such as imprisonment, drug use, and tattooing. The principal way of transmission of hepatitis B is transmission by unprotected sexual intercourse. The Roma population uses protective means, such as condoms, less frequently than the majority population [12].

Risk factors for transmission of hepatitis B in Roma settlements were male gender, older age, imprisonment, and tattooing [52]. Perinatal transmission also plays an important role in the spread of hepatitis B virus infections [53]. Currently, a multitude of therapeutic options exist for the treatment of chronic hepatitis B, in which patients achieve the virologic response desired in the vast majority of cases. However, Roma have less compliance to the treatment of chronic hepatitis B with pegylated interferons than the majority population [54]. The optimal way to prevent the spread of hepatitis B virus in the Roma settlements would be to vaccinate patients who do not yet show serological markers of hepatitis B.

In the HepaMeta study, only three patients from the Roma population had chronic hepatitis C (0.7%). This is attributed to the fact that intravenous drug use was observed in only a small part of the Roma population living in the settlements. A Czech study found a very high prevalence of hepatitis C in socially excluded Roma people using intravenous drugs [55].

In the HepaMeta study, a higher prevalence of hepatitis E antibodies was observed in the Roma population compared to the majority population. This finding cannot be clearly explained. In the Roma population, the availability of sewage systems, water supply, flush toilets, bathrooms, or showers was markedly reduced when compared to the majority population, but there was no significant difference in the incidence of hepatitis E among the Roma who had or did not have access to these sanitary facilities [13].

Roma in settlements had a significantly higher prevalence of antibodies to *Toxoplasma gondii* and *Toxocara* spp. when compared to the majority. These serological findings correlated with clinical manifestations such as muscle pain in both parasitic diseases, and flu-like symptoms, headache, and diarrhea only in *Toxoplasma gondii* infection. A high prevalence of both parasitic diseases can be attributed to the lack of sanitary facilities [15].

The HepaMeta study has several limitations. A major limitation is that both populations were compared only in the 18–55 age range. No information on socio-economic conditions, lifestyle, or morbidity was obtained from the pediatric and geriatric population in the study. Another limiting factor was the relatively small number of patients enrolled in both groups. The cross-sectional design of the study does not allow assessment of morbidity changes and eventually the analysis of mortality of patients in both groups. The main strength of the HepaMeta study was the fact that it was the first to monitor the complex prevalence of diseases and their association with socio-economic status and lifestyle in the Roma population aged between 18 and 55 years.

## 5. Conclusions

In conclusion, a poor socio-economic status, unhealthy lifestyle, and barriers precluding access to healthcare in the Roma population can lead to a higher morbidity in the said population compared to its non-Roma counterpart, as well as an increase in infection rates with hepatitis B and E and some parasitic diseases. The abovementioned findings could contribute to the fact that Roma men have a life expectancy of 7.5 years and Roma women of 6.6 years shorter than the general population [56].

## Figures and Tables

**Table 1:** Characteristics of the Roma and non-Roma population in the HepaMeta study.

Evaluated Variable	Roma	Non-Roma	*p*
Total number	452	403	
Male sex	159 (35.2%)	185 (45.9%)	0.001
Education primary	360 (81.3%)	9 (2.3%)	<0.0001
Education vocational	73 (16.5%)	84 (21.4%)	
Education higher	10 (2.3%)	300 (76.3%)	
Unemployment	396 (89.6%)	102 (26.4%)	<0.0001
Lack of household equipment *	281 (62.2%)	78 (19.4%)	<0.0001
Payment problems **	218 (48.2%)	49 (12.2%)	<0.0001

Variable numbers of missing responses are in each row; thus, the total count does not add to 452 or 403 respondents in Roma and non-Roma, respectively. * An aggregate of the lack of at least one item of the following: sewerage system, water supply, flush toilet, bathroom or shower, electricity supply; ** an aggregate of issues related to paying at least one item of the following: rent, loan, healthcare, energies, other expenses.

## Appendix A

HepaMeta Team members: Peter Jarcuska, Andrea Madarasova Geckova, Mária Marekova, Daniel Pella, Leonard Siegfried, Pavol Jarcuska, Lydia Pastvova, Ján Fedacko, Jana Kollarova, Peter Kolarcik, Daniela Bobakova, Zuzana Dankulincova Veselska, Ingrid Babinska, Sylvia Drazilova, Jaroslav Rosenberger, Ivan Schreter, Pavol Kristian, Eduard Veseliny, Martin Janicko, Ladislav Virag, Anna Birkova, Marta Kmetova, Monika Halanová, Darina Petrasova, Katarína Carikova, Viera Lovayova, Lucia Merkovska, Lucia Jedlickova, and Ivana Valkova.
